# Infectious Diseases and Wildlife Conservation Medicine: The Case of the Canine Distemper in European Wolf Population

**DOI:** 10.3390/ani10122426

**Published:** 2020-12-18

**Authors:** Cristina E. Di Francesco, Camilla Smoglica, Simone Angelucci

**Affiliations:** 1 Faculty of Veterinary Medicine, University of Teramo, Loc. Piano D’Accio, 64110 Teramo, Italy; csmoglica@unite.it (C.S.); sangelucci@unite.it (S.A.); 2 Majella National Park, Caramanico Terme, 65023 Pescara, Italy

Canine distemper is a contagious infectious disease, caused by canine distemper virus (CDV) belonging to *Morbillivirus* genus, *Paramyxoviridae* family, representing a serious threat for domestic and wild carnivores [1]. In Europe, over the last two decades, canine distemper occurred in different wild species highlighting questions about the effective role of wildlife in the maintenance and transmission dynamics of this infection [2,3,4,5]. In particular, in wolf (*Canis lupus*), CDV appears to cause direct mortality in geographically restricted populations even if with different patterns. While in Iberian wolves (*Canis lupus signatus*), the virus was detected mainly in 2005–2008 years, and the serological data suggest a diffuse population-level immunity [6], the Alpine wolf population seems poorly involved in the multiple and severe epidemic events that affected wildlife in Northern Italy since 2006 [7,8]. Conversely, in 2013 another subpopulation of wolves, living in the Apennine region of Central Italy, experienced a single episode of CDVinfection, responsible for the death of at least 30 animals, with potentially dramatic consequences for the conservation of the species [5]. Based on the phylogenetic analysis of the haemagglutinin (H) encoding gene, the virus is currently classified in 17 different lineages or genotypes with different geographical distribution (vaccine strains America-1, America-2, America-3, America-4, America-5 Arctic-like, Rockborn-like, Asia-1, Asia-2, Asia-3, Asia-4, Africa-1, Africa-2, European Wildlife, Europe/South America-1, South America-2, and South America-3) [9]. With respect to the aforementioned outbreaks, the genetic characterization of the viral strains suggests that different epidemiological scenarios may have influenced the evolution of the diseases in wolves. The Europe and Arctic-like lineages, recovered from Iberian and Apennine wolves, respectively, and typically found in domestic dogs, highlighted the domestic origin of the infection. On the contrary, the viral strains spreading in Alpine wildlife became well adapted to the wild carnivores and clustered within the European Wildlife lineage. This group was exclusively recorded in European wild species except for a single domestic spill-over, recently described in an unvaccinated puppy [8,10]. Based on these data, continuous monitoring of viral strains associated to the CDV infection, not only in wild species as European wolf but in domestic animals also, is considered crucial to highlight any genetic variation influencing the host–range and the geographical distribution of the pathogen.

The phylogenetic analysis of the H gene and the related amino acid sequences represents the most common tool for CDV characterization. According to this method, strains showing the protein H amino acid divergence less than 3.5% belong to a unique lineage [11], while at least the 98% of amino acid identity is necessary for the sub-lineages’ identification [12]. Comparing H protein sequences derived from diverse host species, the sites 530 and 549 are the most frequently involved in substitutions potentially related to the host adaptation [13]. Nevertheless, the results obtained until now do not appear to be univocal; the 530G and 549H substitutions, for instance, were considered typical in wild species, but they were reported in domestic dogs, too [10,14,15]. Probably, other mutations in H gene and/or other targets, such as genes encoding for non-structural C and V protein, should be investigated and additional studies including more sequences of CDV strains coming from wild species are necessary.

Not only the genetic determinants in viral RNA are involved in CD occurrence in wild species, but many different factors, in particular everywhere the anthropogenic pressure is more persistent, must be considered. The size and spatial organization of each population (packs home range, relations between packs, movement patterns), any potential overlap with domestic species and human activities, the co-existence of multiple hosts and pathogens require a multidisciplinary approach to study the dynamic of infections [16].

Finally, even if no report of CDV transmission from animals to humans is still available, the adaptation of the virus to non-human primates was demonstrated [17], and the ability to use the human SLAM receptor in Vero cell is related to few mutations in the H gene [18]. Based on these data, a future scenario where the inter-species jump will occur and CDV will acquire the ability to infect the humans cannot be completely ruled out.
